# Biochar Induces Changes to Basic Soil Properties and Bacterial Communities of Different Soils to Varying Degrees at 25 mm Rainfall: More Effective on Acidic Soils

**DOI:** 10.3389/fmicb.2019.01321

**Published:** 2019-06-12

**Authors:** Mengyang Zhang, Muhammad Riaz, Lin Zhang, Zeinab El-desouki, Cuncang Jiang

**Affiliations:** ^1^Microelement Research Center, College of Resources and Environment, Huazhong Agricultural University, Wuhan, China; ^2^Department of Soil Sciences, Faculty of Agriculture, Ain Shams University, Cairo, Egypt

**Keywords:** biochar, chemical fertilizer, different soils, simulated rainfall, bacteria, physicochemical properties

## Abstract

Biochar and chemical fertilizer have been widely used in agriculture. Most studies have proved that they not only alter soil nutrient content, but also have an impact on soil microbial communities. However, the effects of biochar and chemical fertilizer application on the overall bacterial community in different soil types under rainfall conditions are not yet understood. We took rainfall as a fixed influencing factor and selected four typical soils of China to investigate the bacterial effects of biochar and chemical fertilizer at 25 mm rainfall, and to identify specific differential bacteria and their functions, and to explore the changes of the bacterial community structure of different soil types. The depth of simulated rainfall was 25 mm each time. Yellow-brown soil, fluvo-aquic soil, lou soil, and black soil were chosen for experiment and each soil was divided into four treatments, included non-biochar and non-fertilizer (CK), fertilizer alone (F), biochar alone (C), and combination of biochar and fertilizer (FC). The results indicated that biochar and fertilizer have a more significant effect on bacterial communities in acidic soils. The amendment of biochar and fertilizer alone or together identified 3 (*f_Oxalobacteraceae*, *f_Solibacteraceae_Subgroup_3*, *f_Sphingomonadaceae*), 5 (*f_Chitinophagaceae*, *f_Comamonadaceae*, *f_Geobacteraceae*, *f_norank_o_SC-I-84*, *f_norank_c_OPB35_soil_group*), 1 (*f_Blastocatellaceae_Subgroup_4*) and 0 differential bacteria in yellow-brown soil, fluvo-aquic soil, lou soil, and black soil by statistical test. In yellow-brown soil, the application of biochar alone increased the relative abundance of potential pathogens within the *Sphingomonadaceae* and reduced the relative abundance of beneficial bacteria in *Solibacteraceae*, but the addition of biochar and fertilizer together increased the relative abundance of some beneficial bacteria in *Oxalobacteraceae*. In fluvo-aquic soil, both biochar, and chemical fertilizers promoted the relative abundance of some beneficial bacteria belonging to *Chitinophagaceae*, *Comamonadaceae*, and *Geobacteraceae* that may be involved in nutrient cycling, degradation of plant residues and increase of metal tolerance. The interactions between acidic soil bacterial communities and measured soil parameters including pH, organic matter were found to be statistically significant. Results from this study revealed that it is necessary to formulate biochar and fertilizer application schemes based on different soil types.

## Introduction

Chemical fertilizers have been applied in agriculture for a long time in improving soil nutrient contents and ensuring crop growth requirements. However, it is well known that the constant application of fertilizers has various harmful effects on the soil (Adesemoye and Kloepper, 2009). Biochar is a product with high carbon content formed by low-temperature pyrolysis of agricultural waste in the absence of oxygen, and biochar has been suggested to improve soil pH, soil fertility, and crop yield (Lehmann, 2007; Xu et al., 2012; Zeelie, 2012; Shaaban et al., 2018; Yu et al., 2019).

The soil ecosystem includes microorganisms, minerals, organic matter, water, and air in pedosphere. Soil microbes are one of the main components of the soil ecosystem, mainly composed of bacteria, fungi, algae, and a variety of tiny protozoans, and among them, bacteria is the most abundant (Young and Crawford, 2004). In addition, soil microbes are an essential part of soil physical and chemical processes (Schimel and Schaeffer, 2012; Fierer, 2017). Many studies have investigated the effects of biochar and chemical fertilizers on soil bacteria. Most studies suggested that biochar could affect the abundance of microorganisms due to its special structure and nutrient holding capacity or indirectly change the physicochemical properties of the soil (Lehmann et al., 2011; Ameloot et al., 2013; Jaafar et al., 2014). Some previous studies reported that the microbial communities in the soil change differently with long-term and short-term biochar application, and the bacterial diversity in the soil changes significantly in a short-term biochar amendment (Jin, 2010; Khodadad et al., 2011). Yu et al. (2018) proposed that biochar can increase community diversity and complexity in acidic soil through long-term experiments. Through the analysis of previous studies, it was proposed that the effects of biochar on soil microbes are not uniform due to the diversity of biochar and soil types. Similarly, the results of chemical fertilizers on soil microbial communities are not uniform. Some studies suggested that fertilizers could change soil microbial composition, while others did not find significant effect (Kirchmann et al., 2013; Geisseler and Scow, 2014; Geisseler et al., 2016).

A large number of studies have been carried out on the effects of biochar and chemical fertilizers on the soil microbial community. However, few of them have considered rainfall as a fixed influencing factor in experiments to study the effects of biochar and chemical fertilizers on bacteria. Due to the increase of global temperature in recent years, the water vapor contents in the atmosphere changed accordingly, caused variations in the atmospheric water circulation in the world, which eventually led to an increase in global precipitation frequency and an increase in extreme precipitation (Dore, 2005; Zhang et al., 2007; O”Gorman and Schneider, 2009). Soil moisture varies with rainfall, which determines the availability of water for plants and microorganisms (Sorensen et al., 2013). Liu et al. (2010) indicated that the high availability of soil water promoted microbial growth and increased microbial metabolic activity. Ren et al. (2018) demonstrated by a meta-analysis that rainfall significantly affected soil microbial communities. Schimel et al. (2007) argued that rainfall might have an impact on soil microbes to acquire substrates.

According to the China Meteorological Disaster Yearbook, we chose 25 mm in this experiment to simulate daily rainfall (Song, 2015, 2016). We took rainfall as a fixed influencing factor and selected four typical soils of China to investigate the bacterial effects of biochar and chemical fertilizers in different types of soils at 25 mm rainfall, and to identify specific differential bacteria and its functions in the soil, and to explore the changes of biochar and chemical fertilizers on the bacterial community structure of different soil types. Previous studies showed that the ameliorative effect of biochar on acidic soil not only changed the pH but also changed the original structure of the soil (Lehmann, 2007; Cantrell et al., 2012; Novak et al., 2013; Bruun et al., 2014; Gul et al., 2015). Our previous experiment identified that biochar had a significant effect on the bacterial community structure of acidic red soil and the combination of biochar and chemical fertilizers was the most beneficial to the changes in soil bacterial community (Zhang et al., 2019). Based on these findings, we hypothesized that biochar may have a more significant effect on bacterial communities in acidic soils under simulated 25 mm rainfall conditions, but should be quite different for different types of soils. Moreover, we hypothesized that co-application of biochar and fertilizer may be more beneficial to the soil than a single application.

## Materials and Methods

### Site Selection and Experiment Description

In the present study, four types of soils were used: yellow-brown soil (YB), collected from Wuhan, Hubei province; fluvo-aquic soil (M), collected from Qingdao, Shandong province; lou soil (L), collected from Xian, Shanxi province; and black soil (B), collected from Daqing, Heilongjiang province. At each site, the soils were randomly collected from tillage layers (0–20 cm) within an area of approximately 100 m^2^.

In order to transport soil to the laboratory, soil samples were placed in sterile bags, and put it on ice. After arriving at the lab, the soil was placed in a ventilated room for 1 week to get air-dried soil. Finally, the soil samples were composited, thoroughly homogenized and sieved through 2 mm mesh to remove small roots, plant residues, and gravels. The PVC cups were utilized as soil culture medium (Supplementary Figure S1), and the whole experiment was carried out in the key laboratory of soil micronutrient at Huazhong Agriculture University, Wuhan, for 28 days. The experiment contained four treatments, i.e., control treatment (CK), biochar (C) treatment alone (w/w = 2%), chemical fertilizer (F) treatment alone [0.14 g kg^−1^ KH_2_PO_4_, 0.51 g kg^−1^ KNO_3_, 0.80 g kg^−1^ NH_4_NO_3_, and 0.95 g kg^−1^ Ca(NO_3_)_2_] and combined biochar and fertilizer treatment (FC). The biochar was obtained from Shenyang Agricultural University, which was prepared from peanut shells at 400°C. The basic physicochemical properties of biochar are shown in Supplementary Table S1. The soil (300 g soil per cup) was mixed with treatments and then placed into the PVC cup. Each treatment had four independent repetitions, and a total of 64 PVC cups were set up for this experiment.

Simulated rainfall was used in this study and the specific rainfall was determined by consulting the China Meteorological Disaster Yearbook. By analyzing the data of the China Meteorological Disaster Yearbook, we found that the average daily rainfall of the four regions where the soil materials were collected, exceeds 25 mm in about 10 days, but the rainfall in each region was usually different, such as the daily rainfall in Wuhan is often higher than 25 mm, while the rainfall in Daqing is seldom higher than 25 mm (Supplementary Figure S2). In this research, we chose 25 mm rainfall, because it is more likely the actual rainfall of the four locations where the soil samples were collected.

In order to more realistically simulate the effects of rainfall on the soil, we drilled holes in the bottom of the PVC cup and laid two layers of filter paper to prevent soil loss. After loading the mixed soil, biochar and fertilizer, a layer of filter paper was placed on the surface of the soil to reduce the disturbance of soil surface during simulated rainfall. After the arrangement of the experimental system, deionized water was added to the soil until the water was slightly exuded at the bottom of the PVC cup, followed by 3 days of standing pre-culture. After the pre-culture, the simulated rainfall was applied at 25 mm each time. The interval between consecutive two rainfalls was 2 days, and a total of eight rainfalls were simulated. The experiment was terminated on the third day after the last simulated rainfall. At the end of the experiment, each treatment was randomly selected for three replicates for subsequent analyses. The samples were equally divided into two parts: one part was frozen at −80°C for DNA extraction and another part was preserved at 4°C for further analysis.

### Measurement of Soil Physicochemical Properties and Enzyme Activities

The soil physicochemical properties were determined by the routine method (Bao, 2000) and enzyme activities were assessed as described by Guan (1986). Briefly, for the estimation of urease activity, 5 g of soil after sieving was treated with 10 mL of 10% urea solution, three drops of toluene and 20 mL of citrate buffer (pH = 6.7) and incubated at 37°C for 24 h. After filtration, 3 mL of filtrate was added with 20 mL of water, 4 mL of phenol solution, and 3 mL of sodium hypochlorite solution followed by continuous shaking for 20 min. Subsequently, it was diluted to 50 mL, and urease activity was measured by a microplate spectrophotometer. The sucrase activity was measured by employing 5 g of soil (<2 mm), and 15 mL of sucrose solution, 5 mL phosphate buffer solution (pH = 5.5) and five drops of toluene and incubated at 37°C for 24 h. The soil was filtered, and 1 mL of filtrate was treated with 3 mL of nitro salicylic acid and incubated in boiling water bath for 5 min, then diluted to 50 mL after cooling, and sucrase activity was measured determined by microplate spectrophotometer. The measurement wavelengths of urease and sucrase were 578 and 508 nm, respectively.

Acid and alkaline phosphatase activities were measured by disodium phenyl phosphate as substrate (Dick et al., 2000). Briefly, 1 g of soil (<2 mm) and five drops of toluene were employed followed by shaking for 15 min. Then, 20 mL of 0.5% disodium phenyl phosphate was added to this solution and incubated at 37°C for 24 h. Next, 40 mL of 0.3% aluminum sulfate solution was employed. After filtration, 3 mL of filtrate was added to 50 mL volumetric flask for the color reaction, finally, acid and alkaline phosphatase were determined by a microplate reader. The measurement wavelengths of phosphatase was 400 nm.

### DNA Extraction, PCR, and Sequencing

Microbial DNA from soil samples was extracted by EZNA^®^ Soil DNA Kit (Omega BioTek, Norcross, GA, United States) according to the manufacturer’s protocols. We used NanoDrop 2000 UV-vis spectrophotometer (Thermo Fisher Scientific, Wilmington, DE, United States) to determine the final concentration and purification of DNA, and 1% agarose gel electrophoresis to check the DNA quality. The V4 hypervariable regions of the bacterial 16S rRNA gene were amplified with primers 515F (5′-GTGCCAGCMGCCGCGG-3′) and 806R (5′-GGACTACHVGGGTWTCTAAT-3′) (Liao et al., 2015). Polymerase chain reactions (PCR) amplification were conducted using the following program: 3 min of denaturation at 95°C, 30 cycles of 30 s at 95°C, 30 s for annealing at 55°C, and 45 s for elongation at 72°C, and a final extension at 72°C for 10 min. PCR reactions were performed in triplicate by employing 20 μL mixture containing 4 μL of 5× FastPfu buffer, 2 μL of 2.5 mM dNTPs, 0.8 μL of each primer (5 μM), 0.4 μL of FastPfu polymerase, and 10 ng of template DNA. The resulted PCR products were extracted and further purified and quantified using QuantiFluor^TM^-ST (Promega, United States) according to the manufacturer’s procedure.

According to the standard protocols by Majorbio Bio-pharm Technology Co., Ltd. (Shanghai, China), purified amplicons were pooled in equimolar amounts and sequenced using the strategies of PE250 on an Illumina MiSeq platform (Illumina, San Diego, CA, United States).

### Processing of Sequence Data

Raw sequences generated through MiSeq paired-end sequencing were merged using FLASH (Magoč and Salzberg, 2011). (i) The reads were truncated at any site receiving an average quality score <20 over a 50 bp sliding window. (ii) Sequences with overlap longer than 10 bp were merged according to their overlap with mismatch no more than 2 bp. (iii) Sequences of each sample were separated according to barcodes (exactly matching) and Primers (allowing two nucleotide mismatching), and reads containing ambiguous bases were removed. We used UPARSE clustering to classify OTUs with ≥97% similarity into the same OTU (Edgar, 2013). Then, we separated the representative sequence of each OTU and used the RDP classifier to determine the taxonomic information (Silva128 database) (Wang et al., 2007).

### Statistical Analysis

All the data of soil physicochemical properties were subjected to the one-way analysis of variance by using SPSS 20.0. According to the minimum sequence number of samples, the sequence of all samples was subsampled. The alpha diversity index of bacteria was evaluated using mother^1^. Student’s *t*-test was used to assess the significant difference in the diversity index between treatment and control. The histogram of the bacterial composition was completed by R software. Multiple group comparison and *post hoc* analysis were performed using R software’s stats package and Python’s scipy package to identify differential bacteria, among them, multiple test correction was used for fdr, and CI calculation method was used for scheffe. In order to determine the specific differences in bacterial community structure, Qiime, Python, and R software were used for principal component analysis and sample hierarchical clustering. Moreover, we used the R software’s vegan package to perform redundancy analysis of the bacterial community and compared the sequenced data with the EggNOG database to obtain the clusters of orthologous groups (COG) functional abundance of the soil. The 16S functional prediction is the standardization of the OTU table by PICRUSt; then, through the corresponding greengene id of each OTU, the COG family information corresponding to the OTU was obtained and the abundance of each COG was calculated. According to the information of the COG database, the descriptive information of each COG and its function can be analyzed from the EggNOG database. Finally, all figures were prepared by Adobe Illustrator CS6.

## Results

### Relationship Between Biochar and Chemical Fertilizers and Basic Physicochemical Properties of the Different Soil Types Under 25 mm Rainfall

After the addition of biochar, chemical fertilizers, and simulated rainfall, the basic physicochemical properties of different soils were changed to different degrees (Table 1). The following data analyses were compared with the control treatment (CK) of each treatment. The amendment of biochar significantly increased the pH of acidic soils (yellow-brown soil and fluvo-aquic soil) by 0.5–1. On the other hand, biochar and fertilizer treatments caused a little difference in the pH value of lou soil. However, the pH of the black soil was significantly decreased by 0.5 units. The main purpose of chemical fertilizers was to increase soil available nutrients. The F treatment significantly increased the alkaline nitrogen content of yellow-brown soil and fluvo-aquic soil, but the effect was different in alkaline soil. F treatment had a little effect on the alkaline nitrogen content of lou soil, while in black soil, the alkali nitrogen content in F, C, and FC was decreased by 9.2, 8.7, and 6.7%, respectively, compared with the control. The effect of available phosphorus content was mainly affected by fertilizer rather than biochar. In addition, we also found that the biochar amendment (C, FC) in all soils significantly increased the available potassium and organic matter content of the soil.

**Table 1:** Effects of single or combined application of biochar and chemical fertilizers on soil basic physicochemical properties.

	pH				AN (mg/kg)				AP (mg/kg)				AK (mg/kg)				OM (g/kg)			
	YB	M	L	B	YB	M	L	B	YB	M	L	B	YB	M	L	B	YB	M	L	B
CK	5.37c	6.05c	8.27a	9.13a	68.25c	40.83c	55.42a	126.58a	49.21b	15.92b	26.99b	6.99c	138.33d	63.00d	276.00d	249.33d	12.80b	8.41b	17.74c	42.70b
F	5.47c	5.76d	8.21b	8.53c	106.17a	59.50a	56.93a	114.92b	52.72ab	22.64a	42.34a	19.82b	292.00b	148.33b	399.67b	358.67b	11.86b	8.09b	18.48c	41.43b
C	6.05b	6.71a	8.27a	8.89b	65.33c	44.33c	57.17a	115.50b	46.53b	13.63b	33.18b	17.60b	210.67c	92.33c	353.00c	311.33c	22.03a	15.97a	30.95a	55.96a
FC	6.38a	6.57b	8.23ab	8.47c	92.52b	49.00b	53.08a	118.07ab	55.69a	22.95a	48.82a	31.73a	337.67a	176.00a	466.67a	456.33a	22.57ab	16.04a	29.08b	56.90a

*Different lowercase letters indicate a significant difference between the treatments of the same soil at P < 0.05. pH, AN, AP, AK, OM, represent pH, alkali nitrogen, available phosphorus, available potassium, organic matter, respectively.*

Biochar and fertilizer had different effects on the activities of some enzymes in the soil (Table 2). We found that biochar and fertilizer mainly affected the enzymatic activity of fluvo-aquic soil, and all enzyme activities were decreased significantly (except for sucrase), ranging from 20 to 69%. Each treatment also had some effects on the enzyme activities of yellow-brown soil and black soil, but almost no effect on lou soil. We also found a significant increase in sucrase activity in yellow-brown soil and black soil, such as FC of yellow-brown soil, F and C of black soil.

**Table 2:** Effects of single or combined application of biochar and chemical fertilizers on soil enzyme activities.

	YB				M				L				B			
	Urease (μg/g)	Sucrase (mg/g)	ACP (mg/g)	AKP (mg/g)	Urease (μg/g)	Sucrase (mg/g)	ACP (mg/g)	AKP (mg/g)	Urease (μg/g)	Sucrase (mg/g)	ACP (mg/g)	AKP (mg/g)	Urease (μg/g)	Sucrase (mg/g)	ACP (mg/g)	AKP (mg/g)
CK	20.72a	7.21b	0.13a	0.13a	38.37a	3.43ab	0.15a	0.15a	57.32a	4.28ab	0.10a	0.12a	73.33a	6.09c	0.07a	0.15a
F	17.85ab	8.06ab	0.15a	0.12b	11.87b	2.36b	0.08c	0.08c	61.04a	4.71a	0.08ab	0.12a	79.12a	7.13ab	0.06ab	0.13b
C	16.08ab	4.35c	0.13a	0.14a	17.36b	3.59ab	0.12b	0.12b	54.22a	3.93ab	0.09a	0.10a	64.73b	7.47a	0.06ab	0.13bc
FC	17.54b	9.15a	0.15a	0.10c	18.23b	4.71a	0.12b	0.12b	58.07a	3.40b	0.06b	0.10a	74.40a	6.47bc	0.05b	0.12c

*Different lowercase letters indicate significant difference between the treatments of the same soil at P < 0.05. Urease, Sucrase, ACP, AKP represent urease, sucrase, acid phosphatase, and alkaline phosphatase, respectively.*

### Relationship Between Biochar and Chemical Fertilizers and α-Diversity of Different Soil Types Under 25 mm Rainfall

We observed 2224127 quality sequences with an average of 37086 sequences per sample for the bacterial 16S, from all samples. The read lengths ranged from 275 to 276 bp, with an average of 276 bp of the bacterial 16S. Rarefaction analyses indicated that the numbers of recorded OTUs generally approached saturation plateaus at 25000 randomly selected sequences for bacterial, which indicated that the data volumes for the sequences were reasonable (Supplementary Figure S3).

**FIGURE 1:**
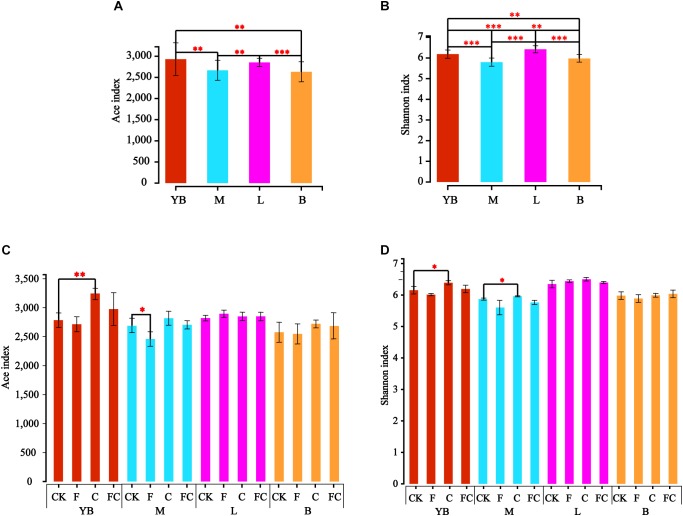
Inter-group difference test for alpha diversity index. **(A,B)** Represent the test results of the Ace index and the Shannon index for different soils. Panels **(C,D)** show the calculation results of the Ace index and the Shannon index between the treatment and the control in the same soil. ^∗∗∗^Correlation is significant at the 0.001 level. ^∗∗^Correlation is significant at the 0.01 level. ^∗^Correlation is significant at the 0.05 level.

We used the Ace and Shannon index to characterize the alpha diversity of soil bacteria (Figure 1). Results demonstrated that there was no significant difference in the Ace index between yellow-brown soil and lou soil, and the same results were obtained for fluvo-aquic soil and black soil (Figure 1A). However, significant differences were found between the four soils in the Shannon index (Figure 1B). More importantly, we found that the trends and ranges of the Ace and Shannon indices were different by biochar and fertilizer in different soils, and the alpha index of acidic soils was changed significantly (Figure 1C,D). Among them, C significantly increased the Ace index in yellow-brown soil while F significantly reduced the Ace index of fluvo-aquic soil, and the amendment of biochar and chemical fertilizer did not cause a significant difference in Ace index between lou soil and black soil. We found that only C significantly increased the Shannon index of yellow-brown soil and fluvo-aquic soil, while the Shannon index of lou soil and black soil was not affected by biochar and fertilizer. The above results indicated that the bacterial diversity of acidic soil was significantly improved after application of by biochar under simulated rainfall, while alkaline soil was almost unaffected.

### Relationship Between Biochar and Chemical Fertilizers and Bacterial Community Composition of Different Soil Types Under 25 mm Rainfall

At the family level, we used the histogram of bacteria to analyze the changes in the composition of four soils by different treatments (Supplementary Figure S4). In order to further clarify the difference of bacteria, we conducted a multi-group comparison in the same soil to find the differential bacteria by one-way analysis of variance. After determining the differential bacteria, we used *post hoc* analysis to compare the treatments with the control to determine significant changes in the relative abundance of bacteria (Figure 3). We found that the amendment of biochar and fertilizer significantly changed the relative abundance of some bacteria in yellow-brown soil and fluvo-aquic soil, but had little effect on lou soil and black soil. These results are consistent with the analysis of alpha diversity.

**FIGURE 2:**
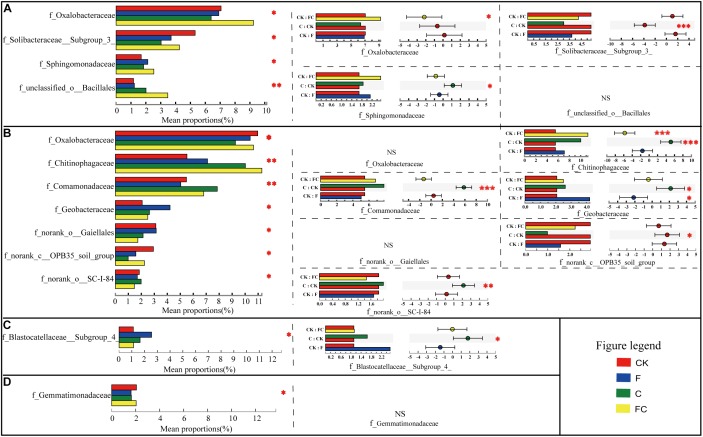
Inter-group difference test of bacterial relative abundance. Multiple group comparison was used by one-way ANOVA. The multiple test calibration and CI calculation methods used fdr and scheffe, respectively, with a confidence interval of 95% and the test range was limited to the first 15 species with relative abundance. The left figure represents the results of multiple group comparisons, with the vertical axis representing the species name and the horizontal axis representing the relative abundance. The right figure shows the *post hoc* analysis, which reflects the significance test results of the differential bacteria between treatment and control. In the left part of *post hoc*, the vertical axis represents the two processing names for comparison, and the horizontal axis represents the relative abundance. The right part represents the proportion of species relative abundance differences within the set confidence interval. **(A–D)** Represent yellow-brown soil, fluvo-aquic soil, lou soil, and black soil, respectively. ^∗∗∗^Correlation is significant at the 0.001 level. ^∗∗^Correlation is significant at the 0.01 level. ^∗^Correlation is significant at the 0.05 level.

We identified four differential families in yellow-brown soil. And through *post hoc* analysis, it was found that three of them had significant differences between treatment and control. The relative abundance of *f_Oxalobacteraceae* in FC was significantly increased by 30.9%, and in *f_Solibacteraceae* subgroup 3 was significantly reduced by 43.0%, while in *f_Spingomonadaceae* increased by 9.4% in C (Figure 2A). Similarly, we determined seven differential families in the fluvo-aquic soil. Five of them had significant differences between treatment and control. In FC, only the relative abundance of *f_Chitinophagaceae* was significantly increased by 104.7%. Similarly, the relative abundances of *f_Chitinophagaceae*, *f_Comamonadaceae*, *f_Geobacteraceae*, *f_norank_o_SC-I-84* were increased by 81.2, 43.4, 26.7, and 7.8%, respectively, however, the relative abundance of *f_norank_c_OPB35* soil group was significantly reduced by 66.4% in C. In addition, the relative abundance of *f_Geobacteraceae* was increased significantly by 103.9% in F (Figure 2B). The results of multiple group comparison showed that only one family had significant change in the lou soil and black soil, and the *post hoc* analysis indicated that the relative abundance of *f_Blastocatellaceae* Subgroup 4 in C was significantly improved by 47.1% in lou soil (Figure 2C,D). In general, the biochar had a greater effect on the soil bacterial community composition than chemical fertilizers, and biochar in acidic soils had a more pronounced effect.

### Relationship Between Biochar and Chemical Fertilizers and β-Diversity of Different Soil Types Under 25 mm Rainfall

**FIGURE 3:**
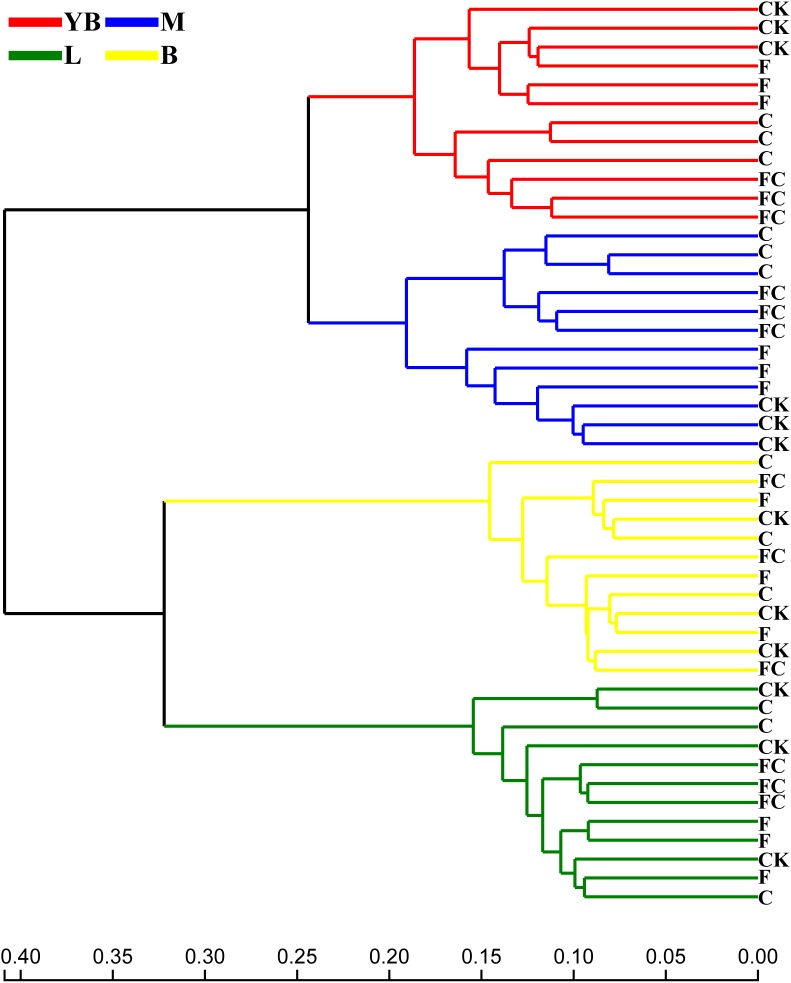
Hierarchical clustering tree on OTU level based on Bray–Curtis. The length of the branches represents the distance between the samples.

**FIGURE 4:**
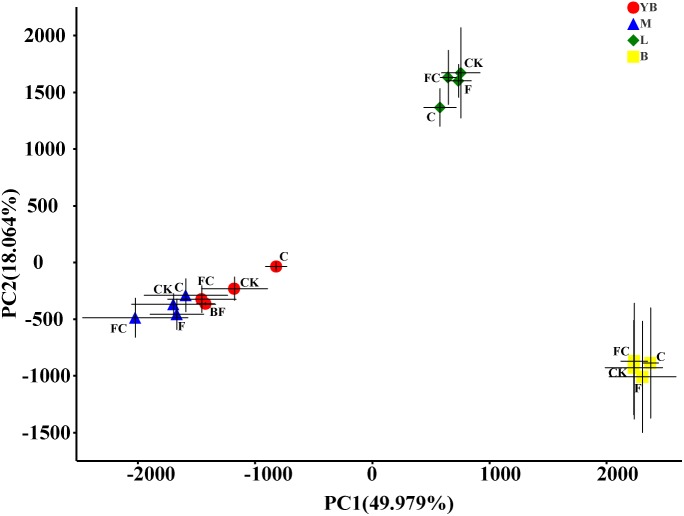
Multidimensional grouping principal component analysis. The points in the figure are the average values of the subgroups on the PC1 and PC2 axes, and the error bar is the standard deviation of the subgroups in the direction of PC1 and PC2.

**FIGURE 5:**
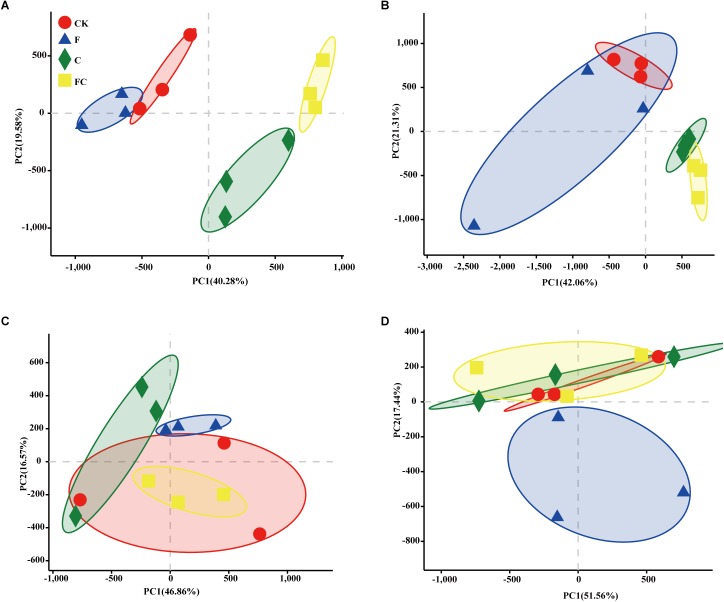
Principal component analysis of each soil. **(A–D)** Represent yellow-brown soil, fluvo-aquic soil, lou soil, and black soil, respectively.

In order to more specifically describe the effects of single or combined amendment of biochar and chemical fertilizers to different soils, we performed clustering and principal component analysis, respectively (Figure 3–5). The hierarchical clustering tree presented two large clusters and four small clusters, of which small clusters represented four soil types, and large clusters characterized acidic soil and alkaline soil. Interestingly, we found a certain pattern in the clustering results of acidic soils. The samples were divided into two clusters in yellow-brown soil and in fluvo-aquic soil, one cluster was CK and F, and the other cluster was C and FC. These results indicated that the amendment of biochar in acid soil had a more significant effect on the bacterial communities compared to single application of chemical fertilizer. On the contrary, the clustering of samples in alkaline soil (lou soil, black soil) was irregular (Figure 3). We used the soil type as first grouping basis and used the treatment as second sub-group basis to perform multi-dimensional group principal component analysis. After biochar and chemical fertilizers and simulated rainfall, the soil bacterial community structure was changed to varying degrees, the change was still not enough to cover the differences in the soil itself (Figure 4). Consistent with the results of the cluster analysis, we found that the biochar in yellow-brown soil and fluvo-aquic soil had a stronger effect on the bacterial community structure, while the single application of chemical fertilizer had almost no effect (Figure 5A,B). However, the biochar and chemical fertilizer alone or in combination did not alter the soil bacterial community structure of lou soil and black soil (Figure 5C,D). ANOSIM analysis also showed that biochar and chemical fertilizers caused significant changes in acidic soils, but had no significant effect on alkaline soils (Table 3).

**Table 3:** Analysis of similarities (ANOSIM) of the amplified 16S rRNA gene fragments of the bacterial community in the same soil.

Soil type	Statistic	*p*-Value	Permutation number
Yellow-brown soil	0.821	0.001	999
Moist soil	0.4877	0.004	999
Lou soil	0.1296	0.232	999
Black soil	−0.2191	0.963	999

### Relationship Between Changes in Basic Physicochemical Properties and Bacterial Community Structure of Acidic Soils Under 25 mm Rainfall

**FIGURE 6:**
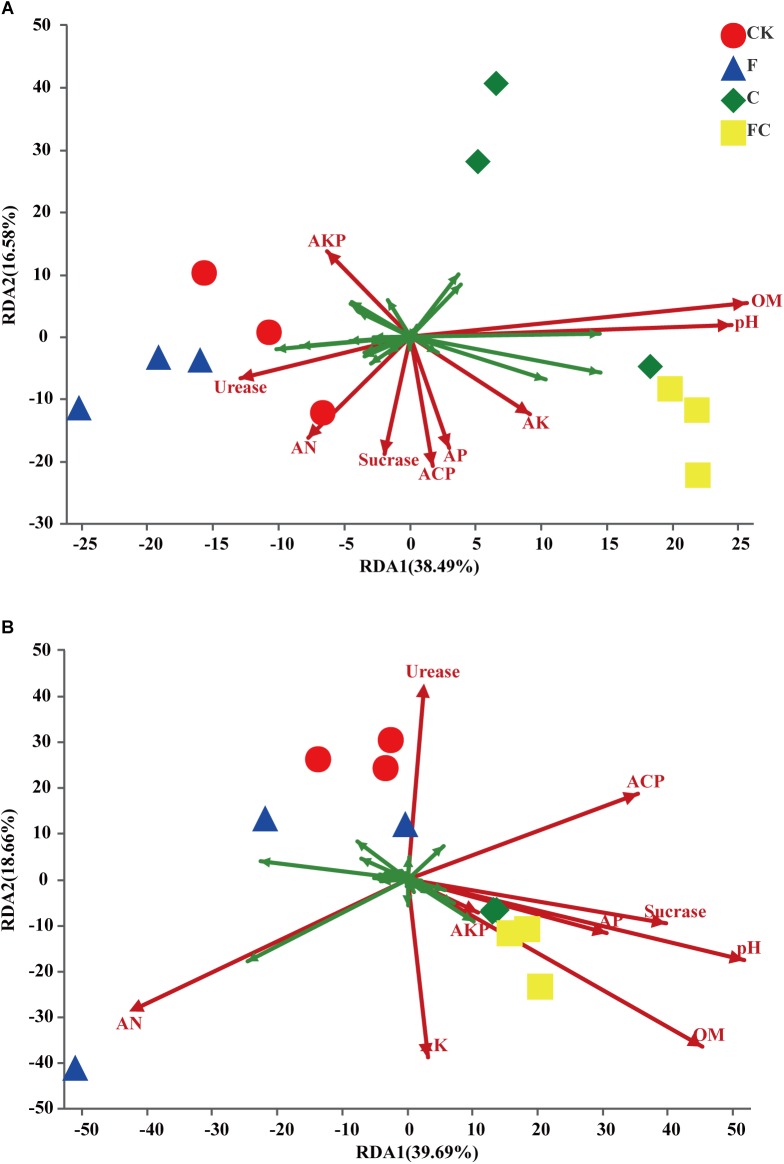
Results of redundancy analysis of acidic soils. **(A,B)** Represent yellow brown soil and fluvo-aquic soil, respectively. The figure shows the top 30 OTUs with high relative abundance. The green arrow represents the species and red arrow represents the environmental factor. pH, AN, AP, AK, OM, Urease, Sucrase, ACP, AKP represent pH, alkali nitrogen, available phosphorus, available potassium, organic matter, urease, sucrase, acid phosphatase, and alkaline phosphatase in two soils, respectively.

The above data analysis showed that the biochar and chemical fertilizers alone or in combination had a significant effect on the bacterial community structure of acidic soils, but had little effect on alkaline soils. More importantly, we determined physicochemical properties of the acidic soil that were changed by biochar and fertilizer, and led to changes in the bacterial community of the acidic soil. We combined the measured soil basic properties and enzyme activities to determine the major environmental factors affecting soil bacterial communities through redundancy analysis (Figure 6). In yellow-brown soil, the bacterial community was mainly affected by pH and organic matter (Figure 6A). The results showed a significant correlation between bacterial community structure of yellow-brown soil and pH, organic matter content and acid phosphatase activity, and the correlation with pH and organic matter content was stronger (Table 4). In the fluvo-aquic soil, the contents of alkaline nitrogen and organic matter, pH, and acid phosphatase activity had the greatest influence on soil bacterial community changes (Figure 6B). And in fluvo-aquic soil, the changes of the bacterial community were significantly correlated with pH, the contents of alkali nitrogen and organic matter and the correlation with pH and organic matter content were stronger (Table 4).

**Table 4:** Datasheet for environmental factors in RDA.

Factor	Yellow-brown soil				Fluvo-aquic soil			
	RDA1	RDA2	*r*^2^	*p*-Value	RDA1	RDA2	*r*^2^	*p*-Value
pH	0.9925	0.1221	0.7767	0.006	0.9353	−0.3539	0.8417	0.001
AN	−0.4768	−0.879	0.4166	0.097	−0.8633	−0.5047	0.6596	0.007
AP	0.1197	−0.9928	0.3942	0.086	0.9233	−0.3842	0.3029	0.178
AK	0.5958	−0.8031	0.281	0.239	0.159	−0.9873	0.3895	0.08
OM	0.9704	0.2416	0.8951	0.003	0.79	−0.613	0.9596	0.001
Urease	−0.8836	−0.4682	0.2808	0.215	−0.0125	0.9999	0.4595	0.079
Sucrase	−0.1606	−0.987	0.4439	0.08	0.9603	−0.2788	0.4671	0.053
ACP	0.0322	−0.9995	0.5257	0.033	0.9165	0.4	0.4036	0.083
AKP	−0.4056	0.914	0.2697	0.236	0.8333	−0.5529	0.048	0.786

*pH, AN, AP, AK, OM, Urease, Sucrase, ACP, AKP represent pH, alkali nitrogen, available phosphorus, available potassium, organic matter, urease, sucrase, acid phosphatase, and alkaline phosphatase, respectively.*

### Relationship Between Biochar and Chemical Fertilizers and Bacterial Functional Diversity of Different Soil Types Under 25 mm Rainfall

In order to study the effects the biochar and chemical fertilizers alone or in combination on the function of different soil bacteria, predictive analysis of the function of soil bacteria was conducted. We selected the top 10 COG functions for analysis. One-way analysis of variance on the top 10 functions was performed in Supplementary Figure S5. The results showed that the effects of biochar and fertilizer treatment on the main abundance of COG in the soil were different depending on the soil type. F-treatment did not significantly change COG functional abundance in fluvo-aquic soil, but biochar treatment (C, FC) significantly reduced the COG functional abundance (Supplementary Figure S5a). In general, biochar and fertilizers changed the COG functional abundance of fluvo-aquic soils but had little effect on yellow-brown soil, lou soil, and black soil (Supplementary Figure S5).

## Discussion

The simulated 25 mm rainfall was determined after reviewing the latest Meteorological Disaster Yearbook, which is in line with the actual situation of sampling points. In the present study, the results showed that the amendment of biochar significantly increased the pH of acidic soils in a short period of time. Biochar had no effect on the pH of the lou soil, but significantly decreased the pH of the black soil (Table 1), which may be related to background pH value of biochar and soil. The background pH values of lou soil, black soil, and biochar used in this experiment were 9.13, 8.27, and 8.76, indicating that the soils with a higher background pH value than biochar either had no effect or decreased the pH. This study demonstrated that the biochar significantly increased the content of available potassium and organic matter in the soil (Table 1), which also verified the results of previous studies (Novak et al., 2009; Kimetu and Lehmann, 2010; Kuzyakov et al., 2014; Liang et al., 2014; Yin et al., 2014). Biochar can adsorb soil organic molecules and promote organic molecule polymerization to form organic matter through surface catalytic activity (Liang et al., 2010; Van Zwieten et al., 2010). In addition, the slow decomposition of biochar contributes to the development of humus and promotes soil fertility (Kimetu and Lehmann, 2010). The increase of available potassium in soil may also be due to the interaction and reaction of biochar with soil in the short term, such as adsorption and desorption, dissolution, precipitation and redox reactions (Joseph et al., 2010). We found that biochar had little effect on the content of alkali nitrogen and available phosphorus (Table 1), which may be due to the low content of mineral nitrogen and available phosphorus in peanut shell biochar. Previous studies have proved that the contents of mineral nitrogen and available phosphorus in some plant biochar were extremely low (Yamato et al., 2006; Rondon et al., 2007; Chan et al., 2008).

In the present study, four common enzyme activities were determined, and results showed the biochar and chemical fertilizer alone or in combination had reducing effect on enzyme activities in fluvo-aquic soil, and also had some effects on yellow-brown soil and black soil, but had almost no effect on lou soil (Table 2). Bailey et al. (2011) pointed out that the special structure and adsorption capacity of biochar determines its complex influence on soil enzyme activity. On the one hand, the reactants may be adsorbed and aggregated by biochar, which promotes enzymatic reaction and leads to the increase of some enzymatic activities. On the other hand, biochar may adsorb enzymatic molecules and conceal the binding sites of enzymatic reaction, which results in the inhibition of some enzymatic reactions. Different studies found that the effect of biochar on soil enzyme activity is completely different and varies with soil type (Bailey et al., 2011; Wu et al., 2013; Oleszczuk et al., 2014). In addition, we supposed that inhibition of microbial growth by chemical fertilizers may be one of the reasons for the decline in the activity of fluvo-aquic soil enzymes. Our results showed that chemical fertilizer alone not only significantly reduced the enzymatic activity of the fluvo-aquic soil, but also significantly decreased its ACE index. Yu et al. (2013) proposed that inorganic fertilizers inhibit the growth of soil microbes. These results also indicate that the effects of biochar and fertilizer on soil enzyme activity vary with soil type. It is worth noting that the incubation time was short, therefore, effects of biochar were short-term, and, however, long-term effects still need further exploration. We carried out simulated rainfall, which possibly affected the enzyme activities in the soil, but the specific mechanism under biochar and fertilizer conditions also needs investigation in the future.

From the results of α-diversity, we indicated that biochar and chemical fertilizer mainly affected acidic soils rather than alkaline soils (Figure 1). This may be due to the conversion of soil from acidity to neutrality by biochar in acidic soils. Some studies have shown that soil bacterial community changes are directly related to pH (Rousk et al., 2010), and acidic conditions inhibit bacterial growth (Padan et al., 2005). The single application of chemical fertilizer led to a significant increase in soil acidity in the fluvo-aquic soil, which may be one of the reasons for reducing the Ace index. Moreover, although we found that the pH changes by biochar and fertilizer in alkaline soil, it was undeniable that the pH after treatment was still strongly alkaline.

From the results of β-diversity, we demonstrated that biochar had a significant effect on the bacterial community structure of the acidic soil (Figure 5), which may be due to the positive effect of biochar on acidic soils. Many studies have demonstrated that biochar as an acid soil improver not only increases soil pH and nutrient content, but also provides shelter for microbial growth (Gheorghe et al., 2009; Kolb et al., 2009; Rousk et al., 2010; Van Zwieten et al., 2010; Jeffery et al., 2011; Yuan and Xu, 2011). The selected lou soil and black soil were alkaline soils in this experiment. Although the black soil showed a significant decrease in pH by biochar, but it was still a relatively alkaline soil. Therefore, biochar had almost no effect on the bacterial community structure of alkaline soil.

Interesting, our results indicated that there was a certain change in the F treatment through α-diversity and β-diversity analysis. For instance, the ace index of the fluvo-aquic soil was significantly reduced (Figure 1) and the F treatment point of each soil can be clearly distinguished from the CK point in the PCA (Figure 5). Many studies also suggested that fertilizer addition shifted microbial communities, decreased enzyme activity, but not affected species richness (Ramirez et al., 2010, 2012; Fierer et al., 2012). Lu et al. (2011) showed that fertilizer application for 5–10 years had the greatest reduction in soil microbial biomass, but Geisseler and Scow (2014) suggested that fertilizer treatment for more than 20 years significantly increased soil microbial biomass. The results of the long-term trials need further research, and this study was under the condition of simulated rainfall, which was different from previous studies, so the long-term results are highly unpredictable. The result indicated that chemical fertilizers may have a more significant effect on acidic soils, and the degree of the specific impact on soil bacterial communities may also be related to the experimental culture time.

The effect of single or combined application of biochar and chemical fertilizer had a much higher effect on acid soil bacteria than alkaline soils. We need to determine what roles these altered bacteria play in the soil. Some studies showed that *Oxalobacteraceae* is involved in soil nitrogen fixation and carbon and sulfur cycles (Favet et al., 2013; Jia et al., 2018). *Solibacteraceae* has been shown to associate with the resistance of some fungal pathogens (*Fusarium oxysporum*) (Mendes et al., 2017) and its relative abundance increases as water content increases (Barnard et al., 2013). Moreover, it has been found to involve in the carbon cycle of the soil (Zhang et al., 2017). *Sphingomonadaceae* is a potential pathogen, because it is usually found in diseased soil areas (Buonaurio et al., 2002; Sanguin et al., 2009; Li et al., 2018). From the results of yellow-brown soil (Figure 2A), the amendment of biochar alone increased the relative abundance of potential pathogens within the *Sphingomonadaceae* and reduce the relative abundance of beneficial bacteria in *Solibacteraceae*, but the application of biochar and fertilizer together improved the relative abundance of some beneficial bacteria in *Oxalobacteraceae* possibly by promoting nutrient cycling. In the fluvo-aquic soil, we identified five bacteria that showed significant differences after treatment (Figure 2B), of which *Chitinophagaceae* and *Comamonadaceae* were found to be ubiquitous in various environments around the world and their relative abundance increased by fertilizer (Delgado-Baquerizo et al., 2018). In addition, the former can also degrade polysaccharides and cellulose (Bernard et al., 2012; Estendorfer et al., 2017; Louca et al., 2017) and the later contains a large number of bacteria that promote metal tolerance and plant growth (Belimov et al., 2005; Piotrowska-Seget et al., 2005; Lin et al., 2016; Chen et al., 2018). *OPB35 soil group* belongs to *Verrucomicrobia*, which is an oligotrophic bacterium, suitable for growth in environments with low nutrient availability (Fierer and Jackson, 2006). The vast majority of the dissimilatory iron-reducing bacteria belong to *Geobacteraceae* (Holmes et al., 2002). Many reports indicated that dissimilatory Fe (III) reduction significantly affects the cycle of carbon, nitrogen, and sulfur, as well as the degradation of organic contaminants and greenhouse gas emissions (Snoeyenboswest et al., 2000; Holmes et al., 2002; Yuan et al., 2016). At present, there are few reports on *SC-I-84*, and the specific functions still need to be explored. According to the results of fluvo-aquic soil (Figure 2B), both biochar and chemical fertilizers can promote the relative abundance of some beneficial bacteria belonging to *Chitinophagaceae*, *Comamonadaceae*, and *Geobacteraceae* that may be involved in nutrient cycling, degradation of plant residues and increase of metal tolerance. In lou soil and black soil (Figure 2C,D), biochar and chemical fertilizer alone or in combination had little effect on soil bacterial communities. One type of bacteria (*Blastocatellaceae*) with significant differences were identified in the lou soil, belonging to the *Acidobacteria*, and is oligotrophic (Pascual et al., 2015; Wang et al., 2016). In black soil, biochar and chemical fertilizers had little effect on soil bacterial community structure.

Our results showed that there was a significant correlation between the soil organic matter content and the bacterial community changes in the acid soil (Figure 6 and Table 4). One reason for this phenomenon may be that biochar is a substance with a high carbon content, which may significantly increase the soil organic matter content after application to the poor organic matter soil. Soil organic matter is an important source of microbial nutrients and an important indicator of soil fertility (Powlson et al., 1987; Bremer et al., 1994; Lovell et al., 1995; Griffiths et al., 1998; Cookson et al., 2005). Liu et al. (2015) proposed that organic matter content is the main influencing factor affecting soil fungal community structure rather than bacteria. Jiao et al. (2011) indicated that there was a positive correlation between the content of organic matter and bacterial biomass. Many studies indicated that decomposable carbon component present in biochar can be utilized as a carbon source by microorganisms (Zimmerman et al., 2011; Troy et al., 2013), which promotes the mineralization of biochar and provides energy for the growth of microorganisms. Enhanced microbial activity promotes the humification of organic matter, resulting in more complex and stable organic compounds. In addition to pH and organic matter content, the activity of acid phosphatase in yellow-brown soil and alkali nitrogen content in fluvo-aquic soil was also significantly correlated with the changes of soil bacterial communities (Figure 6 and Table 4). This indicates that biochar and chemical fertilizers alone or in combination can significantly change the bacterial community structure of acid soils, but different types of soils alter the bacterial community structure by changing different physicochemical properties. In addition, we also found that the amendment of biochar reduced the COG functional abundance of fluvo-aquic soil but had little effect on the other three soils (Supplementary Figure S5). This result indicated that although biochar significantly changes the bacterial community structure of acidic soil, there are still differences in the effects on different types of acidic soils.

## Conclusion

With experiencing 25 mm simulated rainfall with the single or combined application of biochar and fertilizer in the soil, biochar can still improve the acid soil by increasing pH and nutrient contents. In addition, biochar and chemical fertilizer have much higher effects on acid soil bacterial communities than alkaline soils, but the effects on different types of acidic soils are completely different. In yellow-brown soil, the amendment of biochar alone increased the relative abundance of potential pathogens within the *Sphingomonadaceae* and reduced the relative abundance of beneficial bacteria in *Solibacteraceae*, but the application of biochar and fertilizer together improved the relative abundance of some beneficial bacteria in *Oxalobacteraceae* possibly by promoting nutrient cycling. In fluvo-aquic soil, both biochar and chemical fertilizers promoted the relative abundance of some beneficial bacteria belonging to *Chitinophagaceae*, *Comamonadaceae*, and *Geobacteraceae* that may be involved in nutrient cycling, degradation of plant residues and increase of metal tolerance. The interactions between acidic soil bacterial communities and measured soil parameters including pH, organic matter were found to be statistically significant. We demonstrated that the combination of biochar and fertilizer was not suitable for all soils. In other words, it is necessary to formulate biochar and fertilizer addition schemes based on different soil types.

## Data Availability

This manuscript contains previously unpublished data. The name of the repository and accession number are not available.

## Author Contributions

MZ designed the experiments. LZ maintained the experimental process and determined soil physiochemical properties. MR, ZE-D, and CJ revised the manuscript grammatically. All authors read and approved the final manuscript.

## Conflict of Interest Statement

The authors declare that the research was conducted in the absence of any commercial or financial relationships that could be construed as a potential conflict of interest.

## Footnotes

1 http://www.mothur.org/wiki/Schloss_SOP#Alpha_diversity

## Supplementary Material

FIGURE S1 The structure diagram of the culture device.

FIGURE S2 Distribution map of annual average rainfall days (≥25 mm or 50 mm). **(a)** is the distribution of the average annual rainfall days ≥25 mm in China in 2014. **(b)** is the distribution of the average annual rainfall days ≥50 mm in China in 2014. **(c)** is the distribution of the average annual rainfall days ≥25 mm in China in 2015. **(d)** is the distribution of the average annual rainfall days ≥50 mm in China in 2015. As the color deepens, the number of days gradually increases. Data from the China Meteorological Disaster Yearbook (Song, 2015, 2016).

FIGURE S3 Rarefaction curve of all soil sample.

FIGURE S4 Histogram of bacterial community composition at the family level. **(a–d)** represent yellow-brown soil, fluvo-aquic soil, lou soil, and black soil, respectively.

FIGURE S5 Analysis of variance of COG function between treatments in each soil. The **(a–d)** in the upper left corner of each picture represent yellow-brown soil, fluvo-aquic soil, lou soil and black soil. Different lowercase letters on the same color represent significant differences between treatments at *p* < 0.05 level.

TABLE S1 Basic physicochemical properties of biochar.
