# Supplementary figures and images for: Biochar Induces Changes to Basic Soil Properties and Bacterial Communities of Different Soils to Varying Degrees at 25 mm Rainfall: More Effective on Acidic Soils
Source: Front Microbiol. 2019 Jun 12;10:1321. doi: 10.3389/fmicb.2019.01321 (PMC6582450; doi:10.3389/fmicb.2019.01321)

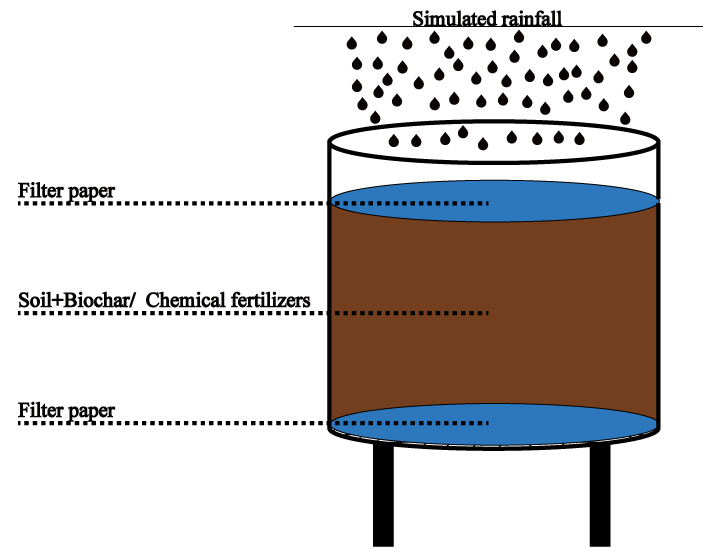

Supplement: FIGURE S1 — The structure diagram of the culture device. [file Image_1.TIF]

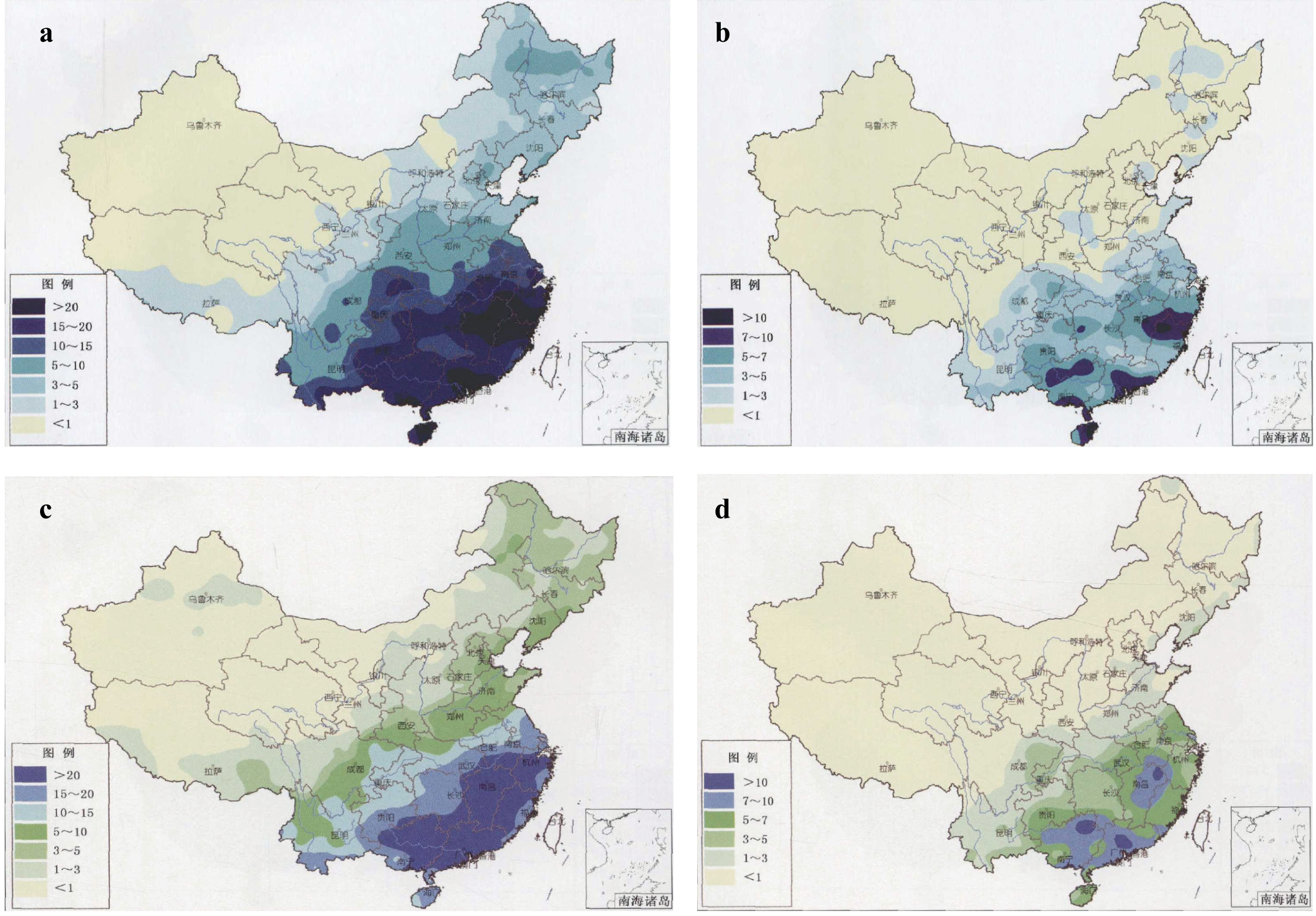

Supplement: FIGURE S2 — Distribution map of annual average rainfall days (≥25 mm or 50 mm). (a) is the distribution of the average annual rainfall days ≥25 mm in China in 2014. (b) is the distribution of the average annual rainfall days ≥50 mm in China in 2014. (c) is the distribution of the average annual rainfall days ≥25 mm in China in 2015. (d) is the distribution of the average annual rainfall days ≥50 mm in China in 2015. As the color deepens, the number of days gradually increases. Data from the China Meteorological Disaster Yearbook (Song, 2015, 2016). [file Image_2.TIF]

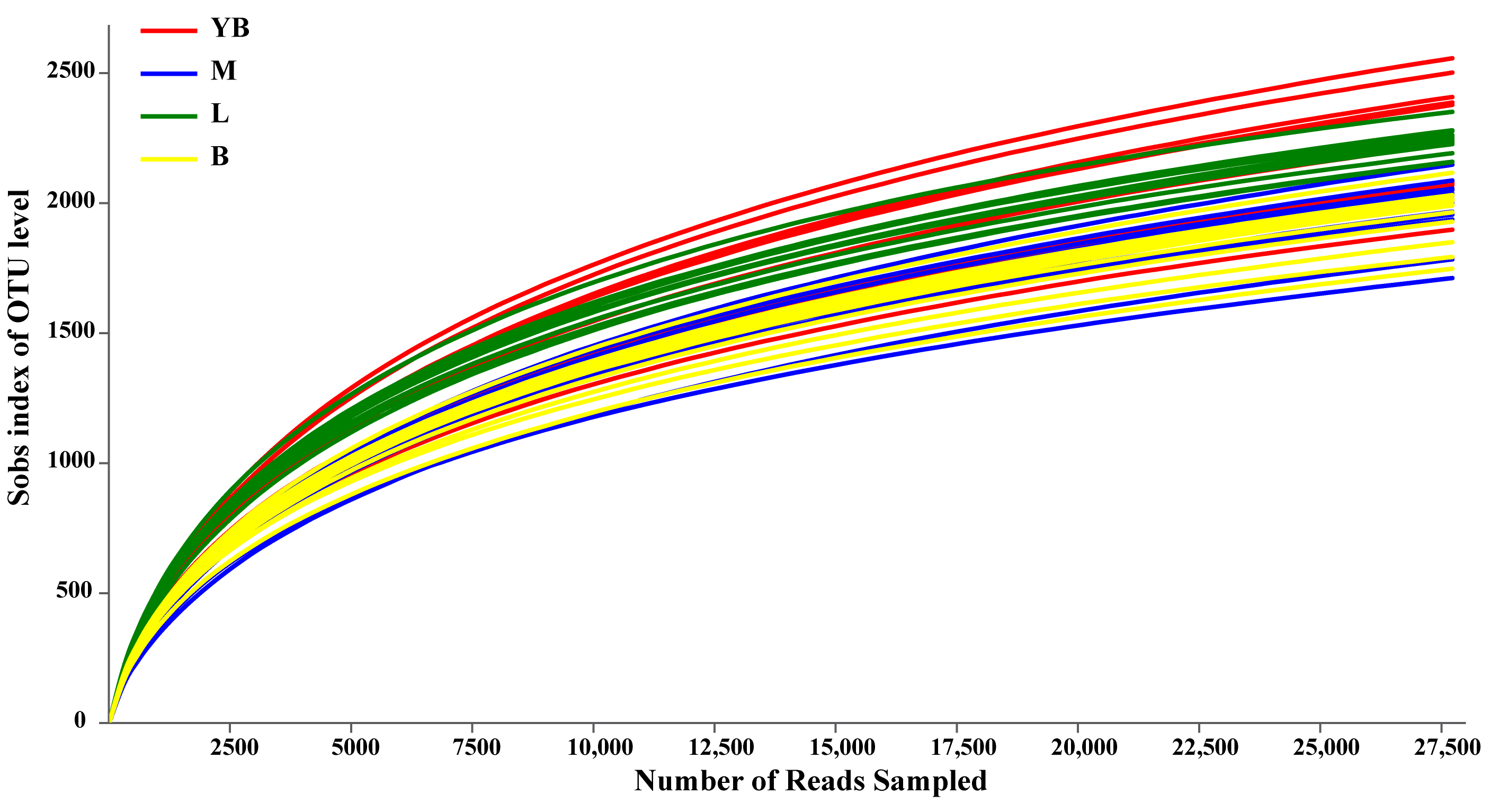

Supplement: FIGURE S3 — Rarefaction curve of all soil sample. [file Image_3.TIF]

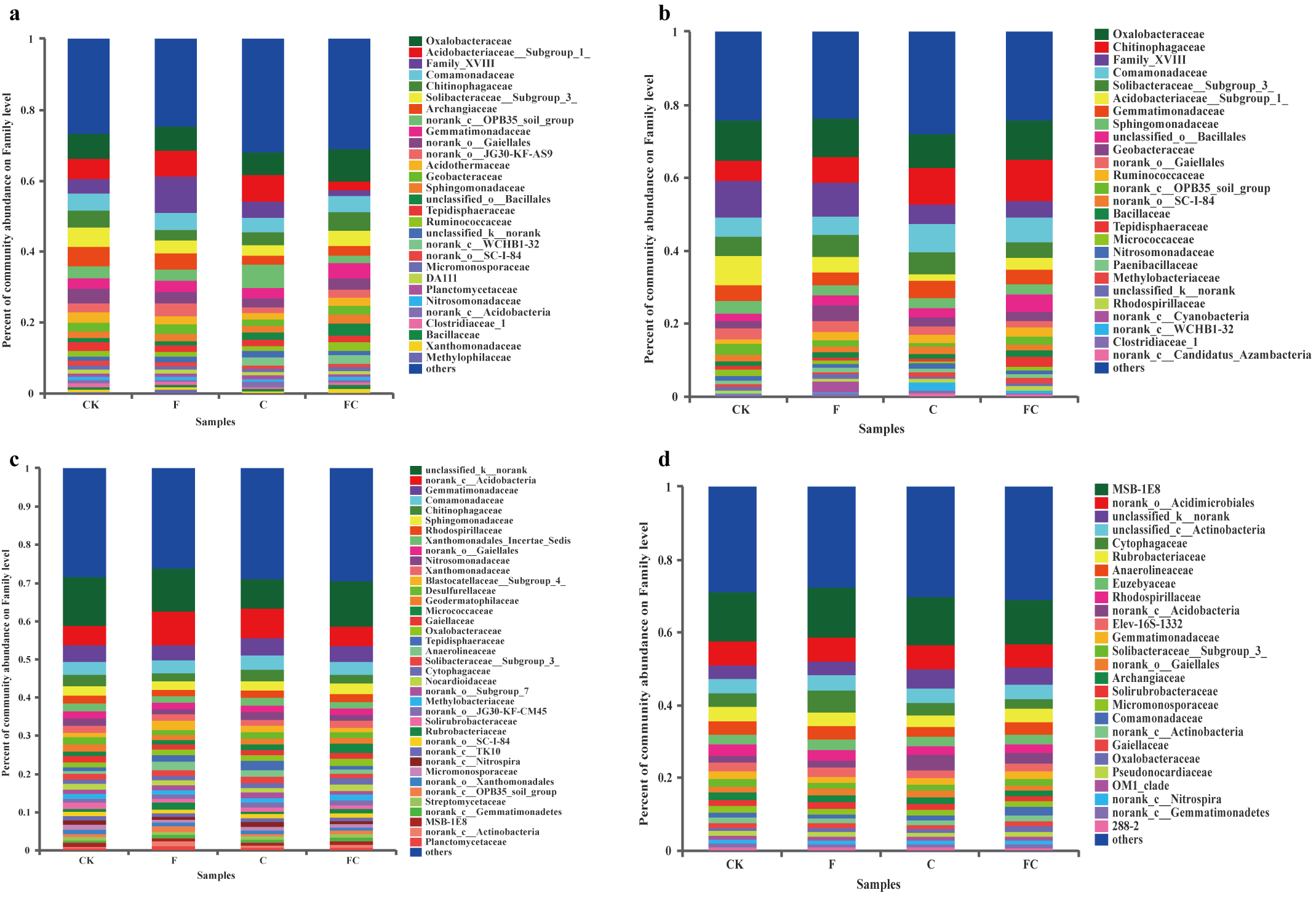

Supplement: FIGURE S4 — Histogram of bacterial community composition at the family level. (a–d) represent yellow-brown soil, fluvo-aquic soil, lou soil, and black soil, respectively. [file Image_4.TIF]

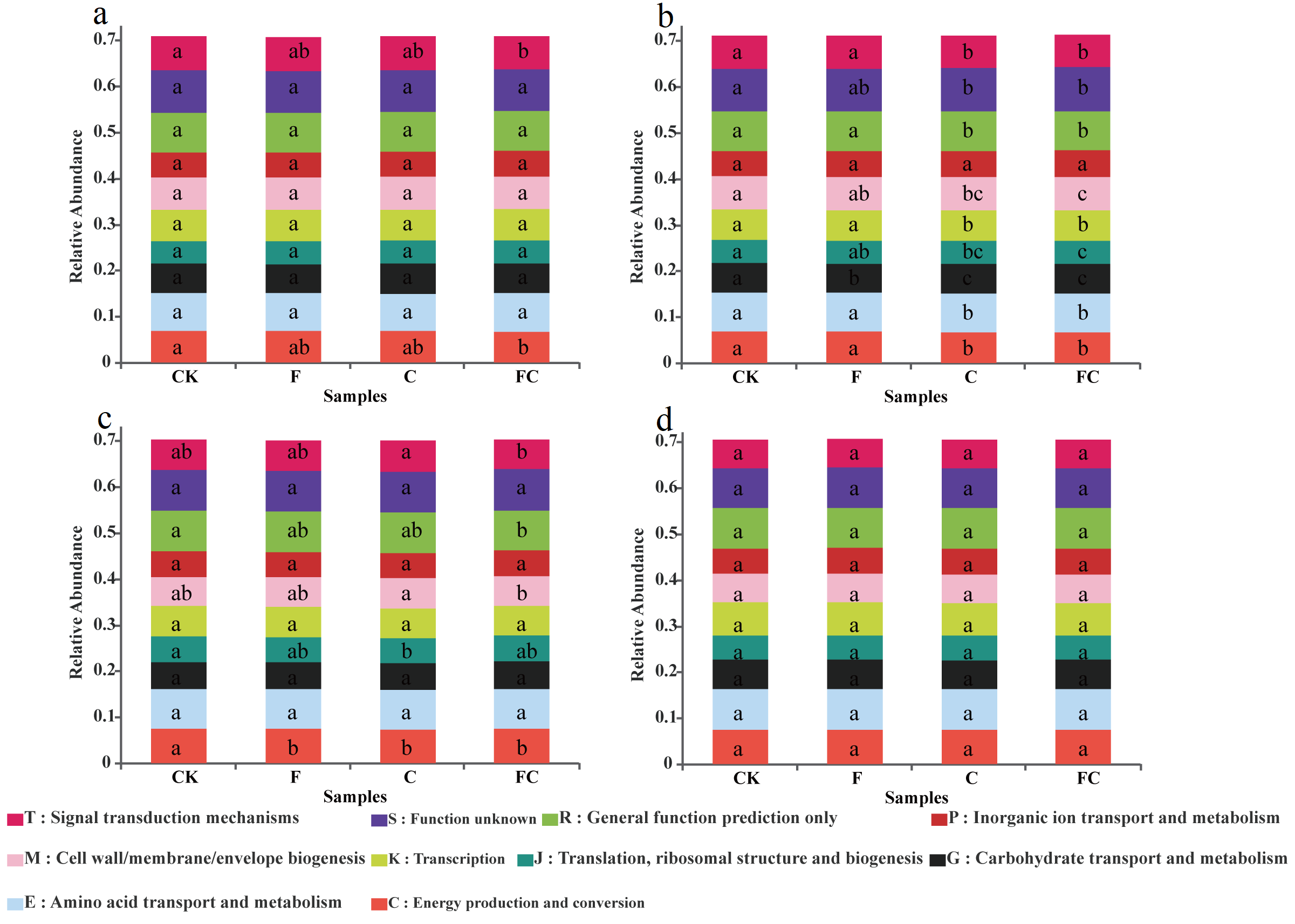

Supplement: FIGURE S5 — Analysis of variance of COG function between treatments in each soil. The (a–d) in the upper left corner of each picture represent yellow-brown soil, fluvo-aquic soil, lou soil and black soil. Different lowercase letters on the same color represent significant differences between treatments at p < 0.05 level. [file Image_5.TIF]
