# Supplementary material for: Biochar Induces Changes to Basic Soil Properties and Bacterial Communities of Different Soils to Varying Degrees at 25 mm Rainfall: More Effective on Acidic Soils
Source: Front Microbiol. 2019 Jun 12;10:1321. doi: 10.3389/fmicb.2019.01321 (PMC6582450; doi:10.3389/fmicb.2019.01321)
Supplement: TABLE S1 — Basic physicochemical properties of biochar. [file Table_1.docx]

**Table S1.** Basic physicochemical properties of biochar

| Index | pH | TN(g/kg) | TP(g/kg) | TK(g/kg) | TC(g/kg) |
| --- | --- | --- | --- | --- | --- |
| Value | 8.76 | 18.84 | 2.59 | 8.48 | 321.93 |

TN, TP, TK, TC represent total nitrogen, total phosphorus, total potassium and total carbon, respectively.
